# Risk of major bleeding in recurrent fallers receiving anticoagulation for atrial fibrillation: a prospective cohort study

**DOI:** 10.1016/j.rpth.2025.102908

**Published:** 2025-05-29

**Authors:** Jean Regina, Fiona Girod, Pedro Marques-Vidal, Alessandra Pia Porretta, Julien Vaucher, Daryoush Samim, Nicolas Rodondi, Elisavet Moutzouri, Jürg H. Beer, Matthias Schwenkglenks, Peter Ammann, Michael Kühne, David Conen, Marie Méan, Swiss-AF investigators

**Affiliations:** 1 Division of Internal Medicine, Lausanne University Hospital, Lausanne, Switzerland; 2 Faculty of Biology and Medicine, University of Lausanne, Lausanne, Switzerland; 3 Service of Cardiology, Heart-Vessels Department, Lausanne University Hospital, Lausanne, Switzerland; 4 Arrhythmic Unit, Service of Cardiology, Hôpital Bichat Claude–Bernard, Assistance publique–hôpitaux de Paris (APHP), Paris, France; 5 Clinical Trial Service Unit and Epidemiological Studies Unit (CTSU), Nuffield Department of Population Health (NDPH), University of Oxford, Oxford, United Kingdom; 6 Department of Cardiology, Inselspital, Bern University Hospital, Bern, Switzerland; 7 Department of General Internal Medicine, Inselspital, Bern University Hospital, University of Bern, Bern, Switzerland; 8 Institute of Primary Health Care, University of Bern, Bern, Switzerland; 9 Department of Medicine, Cantonal Hospital of Baden, Baden, Switzerland; 10 Center for Molecular Cardiology, University Hospital of Zurich, Zurich, Switzerland; 11 Department of Epidemiology, Epidemiology, Biostatistics and Prevention Institute, University of Zurich, Zurich, Switzerland; 12 Department of Public Health, Health Economics Facility, University of Basel, Basel, Switzerland; 13 Division of Cardiology, Kantonsspital St. Gallen, St. Gallen, Switzerland; 14 Division of Cardiology, Department of Medicine, University Hospital Basel, University of Basel, Basel, Switzerland; 15 Cardiovascular Research Institute Basel, University of Basel, Basel, Switzerland; 16 Department of Neurology and Stroke Centre, University Hospital of Basel, Basel, Switzerland; 17 Population Health Research Institute, McMaster University, Hamilton, Ontario, Canada

**Keywords:** anticoagulation, atrial fibrillation, fall, major bleeding, prospective study

## Abstract

**Background:**

The risk of fall-related bleeding is a frequent reason for not following recommendations on anticoagulation in patients with atrial fibrillation (AF).

**Objectives:**

To assess whether patients on anticoagulation therapy with recurrent falls are at an increased risk of bleeding.

**Methods:**

We used data from the Swiss-AF multicenter cohort study, including patients aged ≥65 years with documented AF and oral anticoagulant treatment. Recurrent fallers were defined as those reporting > 1 fall/y. The primary outcome was first major bleeding as defined by the International Society on Thrombosis and Haemostasis. To account for death as a competing event, we used the Fine–Gray competing risk regression model to examine the association between a history of recurrent falls and time to a first major bleeding event. The results were expressed as subdistribution hazard ratios with 95% CIs.

**Results:**

We included 2154 patients (mean age, 73.4 years; 27.5% female), 180 (8.3%) of whom reported recurrent falls. During a median follow-up of 36 months, 368 (17.1%) patients died, and 218 (10.1%) had a first major bleeding event. Recurrent fallers were more likely to experience trauma-related bleeding episodes than nonfallers (16.7% vs 9.2%). The adjusted subdistribution hazard ratio for major bleeding in recurrent fallers was 1.16 (95% CI, 0.74-1.82). In subgroup analyses of patients receiving direct oral anticoagulants or vitamin K antagonists, the risk of major bleeding was not increased for recurrent fallers.

**Conclusion:**

We found no association between recurrent falls and risk of major bleeding in AF patients receiving direct oral anticoagulants or vitamin K antagonists.

## Essentials

• Fall risk is a common reason to avoid anticoagulation in atrial fibrillation.
• This multicenter cohort study examined whether frequent fallers had a higher risk of major bleeding.
• Falls were associated with traumatic bleeding but not with major bleeding.
• These findings support anticoagulation of atrial fibrillation patients with high thromboembolic risk.

## 1 Introduction

Falls occur in 30% to 60% of elderly patients each year, and patients who fall have a higher susceptibility to fall-related injuries than younger patients [[1], [2], [3], [4], [5]]. One out of 6 patients aged >75 years is a recurrent faller, defined as >1 fall in the last year [[6], [7], [8]]. Fear of fall-related bleeding is the most cited reason for not providing anticoagulation to recurrent fallers. Still, it has been estimated that anticoagulation is justified unless a patient falls almost daily, about 295 times per year [9]. Thus, whether recurrent falls increase the risk of bleeding in elderly patients receiving anticoagulants remains a matter of debate. Studies that have specifically addressed this question have been limited by their retrospective design and failure to include clinically relevant nonmajor bleeding as an outcome [10]. Moreover, these studies have been mostly published before the era of direct oral anticoagulants (DOACs) and have found conflicting results [4,10,11].

Atrial fibrillation (AF) is associated with increased mortality and morbidity, particularly stroke [12]. Anticoagulation is a cornerstone in the management of AF but carries a risk of intracranial and major bleeding. Both vitamin K antagonists (VKAs) and DOACs are effective for the prevention of stroke [13]. A meta-analysis of 4 randomized trials comparing the efficacy and safety of DOACs vs warfarin found reductions in both intracranial hemorrhage and major bleeding in patients receiving DOACs [14]. Whether those findings also apply to recurrent fallers receiving DOACs for AF is unknown.

We, therefore, aimed to assess whether recurrent fallers receiving anticoagulation for AF are at an increased risk of bleeding compared with nonrecurrent fallers receiving anticoagulation. We used prospectively collected data from a real-world setting: the Swiss-AF cohort study. Our hypothesis was that if such an association exists, recurrent falls may be identified as a potential modifiable bleeding risk factor, and solutions to prevent and decrease fall risk should be actively implemented.

## 2 Methods

### 2.1 Cohort sample

The study was conducted as part of the Swiss-AF cohort study, a prospective, multicenter, observational cohort study designed to assess long-term medical outcomes in patients with AF from university and nonuniversity hospitals in Switzerland. Consecutive patients aged ≥65 years with (a) paroxysmal AF documented at least twice within the last 24 months, (b) persistent AF, or (c) permanent AF were identified by screening in- and outpatients in the 13 participating hospitals and by contacting general practitioners in the area. Swiss-AF enrolled a total of 2415 patients between 2014 and 2017. A detailed description of the methods has been previously published [15]. Exclusion criteria were (a) inability to provide informed consent, (b) secondary forms of AF, and (c) unavailable follow-up data. Patients with an acute illness within the past 4 weeks were enrolled after resolution of the acute episode. Patients without an assessment of fall history and patients not taking oral anticoagulants (OACs) were excluded.

Patients were followed from cohort inclusion until death, withdrawal, or the study’s conclusion in 2017. Follow-up included yearly face-to-face interviews by trained study personnel in the Swiss-AF cohort and mailed questionnaires. Standardized data collection forms were used by trained study personnel during the clinical visits to collect detailed information on socio-demographic covariates, clinical covariates, concomitant and AF-related treatments, medical interventions, and intercurrent adverse events.

### 2.2 Study outcomes

Our primary outcome was major bleeding defined as: (a) fatal bleeding; (b) bleeding with a reduction in hemoglobin level ≥ 20 g/L within 7 days; (c) bleeding leading to a transfusion of at least 2 units of blood; or (d) symptomatic bleeding in a critical area or organ (intracranial, intraspinal, intraocular, pericardial, intra-articular, intramuscular with compartment syndrome, and retroperitoneal) according to the International Society on Thrombosis and Haemostasis criteria [16].

Secondary outcomes were all-cause death, minor bleeding (defined as any bleeding not meeting the major bleeding definition), traumatic bleeding (defined as bleeding occurring during or immediately after a trauma), and any bleeding (major or minor). Clinical event committees adjudicated all clinical outcomes with 2 independent clinicians who were unaware of other study-specific information and used standardized definitions. A third physician was consulted in case of disagreement.

### 2.3 Main exposure

The risk of falls was assessed in the baseline interviews. We considered patients as recurrent fallers if they answered “yes” to the question, “Did you have recurrent falls?” (ie, > 1/y).

### 2.4 Other covariates

Baseline data on socio-demographics (age and sex - recorded as a binary variable based on biological assignment at enrollment), clinical (body mass index and alcohol abuse) and medical history (any bleeding, stroke or transient ischemic attack, heart failure with preserved or reduced ejection fraction, hypertension, malignancy, and renal failure), concomitant treatments (aspirin and antiplatelet therapy), and AF-related treatments (OAC therapy: DOAC or VKA) were collected by face-to-face interviews. The Congestive heart failure, Hypertension, Age ≥75 years, Diabetes mellitus, Stroke or transient ischemic attack, Vascular disease, Age, and female Sex category (CHA_2_DS_2_-VASc) score was calculated for each patient. Nonfasting venous blood samples were collected from each patient at baseline. Laboratory findings (hemoglobin concentration and platelet count) were collected when available.

### 2.5 Ethical statement

The local ethics committees approved the study. Full decisions can be obtained from the authors upon request. The study was performed in accordance with the Helsinki Declaration and the applicable Swiss legislation. All participants gave their signed informed consent before entering the study.

### 2.6 Statistical analysis

We performed all statistical analyses using STATA 18.0 (Stata Corporation). Descriptive data are presented as absolute numbers (percentages) for categorical variables and as mean (SD) or median value (IQR) for continuous variables.

We examined the association between a history of recurrent falls and time to a first major bleeding using Fine–Gray competing risk regression, accounting for death as a competing event. The rationale for using this model is the substantial death rate in AF patients, with differential death rate between recurrent fallers and nonfallers. By using the Fine–Gray model, we ensured that the impact of recurrent falls on major bleeding risk was not biased by the competing event of death [17]. In addition, we used Cox proportional hazards regression to assess the association between a history of recurrent falls and all-cause mortality [18]. The first multivariable model (model 1) was adjusted according to the variables included in the bleeding risk model derived from the Swiss-AF cohort in 2021 (age ≤75 or >75 years old, history of any bleeding, hypertension, and cancer) [19]. A second multivariable model (model 2), including all variables from model 1 plus sex, renal failure, stroke, and antiplatelet drug therapy, was applied. Results were expressed as subdistribution hazard ratio and 95% CI for Fine–Gray model and as hazard ratio and 95% CI for Cox model. We also performed 2 subgroup analyses, including only patients receiving DOACs or VKAs.

## 3 Results

Of the 2415 patients enrolled in the Swiss-AF study, 31 (1.3%) were excluded because of unavailable follow-up data, and 230 (9.5%) were excluded because they were not anticoagulated. Overall, the excluded patients were similar to included patients, except that excluded patients were more frequently >75 years at enrollment and more frequently presented with previous hypertension (data not shown). The final sample included 2154 patients. The median age was 73.4 years (IQR, 68.4-79.0), and 592 (27.5%) were female. At enrollment, 1215 (56.4%) patients received DOACs, and 938 (43.6%) were on VKA therapy (Table 1). One patient was anticoagulated with deltaparin.

**Table 1:** Patient characteristics by history of recurrent falls.

Characteristic	All	Recurrent faller	
	(*N* = 2154)	Yes (*n* = 180)	No (*n* = 1974)
Age, y	73.4 (68.4-79.0)	77.1 (72.2-82.7)	73.1 (68.2-78.7)
Age >75 y	939 (43.6)	114 (63.3)	825 (41.8)
Female sex	592 (27.5)	72 (40.0)	520 (26.3)
Medical history			
Any bleeding	308 (14.3)	50 (27.8)	258 (13.1)
Stroke	445 (20.7)	57 (31.7)	388 (19.7)
Heart failure	571 (26.5)	75 (41.7)	496 (25.2)
Diabetes	385 (17.9)	47 (26.1)	338 (17.1)
Hypertension	1531 (71.1)	145 (80.6)	1386 (70.2)
Malignancy	340 (15.8)	38 (21.1)	302 (15.3)
Renal failure	457 (21.2)	60 (33.3)	397 (20.1)
Anemia^a^	395 (28.4)	49 (40.8)	346 (27.2)
Low platelets^b^	148 (10.7)	9 (7.6)	139 (11.0)
Alcohol abuse	407 (18.9)	38 (21.1)	369 (18.7)
CHA_2_DS_2_-VASc score	3 (2-5)	4 (3-5)	3 (2-5)
HAS-BLED score	3 (2-4)	3 (2-4)	3 (2-4)
Treatments			
Platelet inhibitors, including aspirin	341 (15.9)	15 (8.3)	326 (16.5)
DOAC therapy	1215 (56.4)	95 (52.8)	1120 (56.7)
VKA therapy	938 (43.6)	85 (47.2)	853 (43.2)
Deltaparin	1 (0.05)	0	1 (0.05)

Results are expressed as median (IQR) or as number of patients (%).

CHA_2_DS_2_-VASc, Congestive heart failure, Hypertension, Age ≥75 years, Diabetes mellitus, Stroke or transient ischemic attack, Vascular disease, Age, and female Sex category; HAS-BLED, Hypertension, Abnormal liver/renal function, Stroke, Bleeding history or predisposition, Labile international normalized ratio, Elderly, Drugs/alcohol concomitantly; DOAC, direct oral anticoagulant; VKA, vitamin K antagonist.

a Defined as hemoglobin < 130 g/L in men and < 120 g/L in women, *n* = 1391.

b Defined as platelets < 150 G/L, *n* = 1384.

### 3.1 Recurrent fallers

Overall, 180 patients (8.3%) were recurrent fallers. When compared with nonrecurrent fallers, recurrent fallers were older, more likely to be female, and had a history of bleeding. They also had more comorbidities, such as stroke, heart failure (with preserved or reduced ejection fraction), hypertension, history of malignancy, and renal failure. The mean CHA_2_DS_2_-VASc score was higher in recurrent fallers than in nonrecurrent fallers (4 [IQR, 3-5] vs 3 [IQR, 2-5]). There were no differences in anticoagulation status and type of anticoagulant according to falling status, but recurrent fallers took fewer platelet inhibitors. Baseline characteristics of participants, stratified by their falling status, are presented in Table 1.

### 3.2 Association between recurrent falls and major bleeding

During a median follow-up of 36 months, 368 (17.1%) patients died, and 218 (10.1%) had a first major bleeding event. The primary and secondary outcomes by history of recurrent falls are shown in Table 2.

**Table 2:** Primary and secondary outcomes by history of recurrent falls.

Outcomes	All	Recurrent faller	
	(*N* = 2154)	Yes (*n* = 180)	No (*n* = 1974)
Major bleeding	218 (10.1)	24 (13.3)	194 (9.8)
Traumatic bleeding	95 (4.4)	11 (6.1)	84 (4.2)
Minor bleeding	294 (13.7)	31 (17.2)	263 (13.3)
Traumatic bleeding	132 (6.1)	22 (12.2)	110 (5.6)
At least 1 traumatic bleeding event	212 (9.8)	30 (16.7)	182 (9.2)
At least 1 bleeding event	458 (21.3)	50 (27.8)	408 (20.7)
Death	368 (17.1)	51 (28.3)	317 (16.1)

Results are expressed as *n* (%). Since participants could experience multiple bleeding events, the number of participants with at least 1 traumatic bleeding event or at least 1 bleeding event does not equal the sum of major and minor bleeding events.

In unadjusted analysis using the Fine–Gray model for bleeding and the Cox model for death, recurrent fallers had a higher risk of death, any bleeding, and traumatic bleeding than nonrecurrent fallers (Tables 3 and 4). After multivariable adjustment of 2 different models, only the increased risk of death and traumatic bleeding among recurrent fallers remained. No association was found between recurrent falls and major or minor bleeding (Tables 3 and 4).

**Table 3:** Association between history of recurrent falls and bleeding, all patients, patients receiving direct oral anticoagulants (*n* = 1215), and patients receiving vitamin K antagonists (*n* = 938).

Outcomes	All (*N* = 2154^a^)	DOAC (*n* = 1215)	VKA (*n* = 938)
	Subdistribution hazard ratio	Subdistribution hazard ratio	Subdistribution hazard ratio
**Major bleeding**			
Unadjusted	1.47 (0.96-2.25)	1.60 (0.85-3.02)	1.32 (0.74-2.35)
Multivariable-adjusted model 1^b^	1.16 (0.74-1.82)	1.04 (0.51-2.10)	1.23 (0.69-2.20)
Multivariable-adjusted model 2^b^	1.06 (0.67-1.68)	0.91 (0.44-1.89)	1.16 (0.64-2.11)
**Minor bleeding**			
Unadjusted	1.38 (0.95-2.00)	1.10 (0.62-1.95)	1.66 (1.01-2.74)
Multivariable-adjusted model 1^b^	1.16 (0.78-1.71)	0.83 (0.45-1.53)	1.54 (0.92-2.57)
Multivariable-adjusted model 2^b^	1.17 (0.78-1.74)	0.83 (0.44-1.55)	1.56 (0.92-2.62)
**Any bleeding**			
Unadjusted	1.49 (1.10-2.00)	1.21 (0.76-1.92)	1.74 (1.18-2.56)
Multivariable-adjusted model 1^b^	1.21 (0.89-1.66)	0.83 (0.50-1.39)	1.63 (1.10-2.42)
Multivariable-adjusted model 2^b^	1.17 (0.85-1.61)	0.78 (0.46-1.32)	1.62 (1.08-2.42)
**Any traumatic bleeding**			
Unadjusted	2.29 (1.54-3.41)	2.23 (1.23-4.03)	2.28 (1.33-3.89)
Multivariable-adjusted model 1^b^	1.82 (1.20-2.77)	1.70 (0.90-3.22)	1.99 (1.15-3.46)
Multivariable-adjusted model 2^b^	1.81 (1.18-2.78)	1.64 (0.84-3.21)	2.04 (1.17-3.57)

Competing risk regression analysis, accounting for death as a competing risk; results are expressed as subdistribution hazard ratio (95% CI).

DOAC, direct oral anticoagulant; VKA, vitamin K antagonist.

a One patient was anticoagulated with deltaparin and not included in the subgroup analysis.

b Multivariable-adjusted model 1 is adjusted for age (<75 or >75 years), history of any bleeding, hypertension, and cancer; model 2 is the same as model 1 plus adjustment for sex, renal failure, stroke, and antiplatelet drug therapy.

**Table 4:** Association between history of recurrent falls and death, all patients, patients receiving direct oral anticoagulants (*n* = 1215), and patients receiving vitamin K antagonists (*n* = 938).

Outcomes	All (*n* = 2154^a^)	DOAC (*n* = 1215)	VKA (*n* = 938)
	Hazard ratio	Hazard ratio	Hazard ratio
**Death**			
Unadjusted	2.18 (1.62-2.94)	2.30 (1.45-3.65)	2.08 (1.41-3.06)
Multivariable-adjusted model 1^b^	1.66 (1.23-2.25)	1.35 (0.83-2.17)	1.92 (1.30-2.84)
Multivariable-adjusted model 2^b^	1.67 (1.23-2.27)	1.27 (0.79-2.07)	2.04 (1.37-3.04)

Results for Cox models are expressed as hazard ratio (95% CI).

DOAC, direct oral anticoagulant; VKA, vitamin K antagonist.

a One patient was anticoagulated with deltaparin and not included in the subgroup analysis.

b Multivariable-adjusted model 1 is adjusted for age (<75 or >75 years), history of any bleeding, hypertension, and cancer; model 2 is the same as model 1 plus adjustment for sex, renal failure, stroke, and antiplatelet drug therapy.

In the subgroup analysis, including 1215 patients receiving DOACs, unadjusted analysis showed that recurrent fallers had a higher risk of death and traumatic bleeding than nonrecurrent fallers; however, these differences disappeared after multivariable adjustment. No association was found between recurrent falls and the other outcomes (Tables 3 and 4).

In the subgroup analysis, including 938 patients receiving VKAs, unadjusted analysis showed that recurrent fallers had a higher risk of minor bleeding, any bleeding, traumatic bleeding, and death than nonrecurrent fallers. The higher risk of traumatic bleeding, any bleeding, and death remained in the multivariable models (Tables 3 and 4).

## 4 Discussion

In this prospective, multicenter cohort study of patients with AF receiving anticoagulants, one-fifth of patients experienced at least 1 bleeding event during follow-up. However, we found no evidence that recurrent fallers on anticoagulant therapy had an increased risk of major bleeding compared with nonrecurrent fallers.

Our findings contradict those of a retrospective study of patients with AF, which reported that prior history of falls, as ascertained from clinical history or medical records, was associated with a 3-fold higher hazard of major bleeding among anticoagulated patients [20]. These findings were limited by the retrospective nature of the study and the small number of patients with a prior history of falls who received anticoagulants (76 patients, 1.1% of the total cohort). Conversely, our results align with an earlier prospective study showing that high risk of falls was not associated with major bleeding in patients treated with OACs [21]. This study primarily included patients treated with VKAs, 60% of whom had AF as an indication for anticoagulation. Our study confirms these findings in patients treated with DOACs for AF.

We also found that cardioembolic risk, as reflected by the CHA_2_DS_2_-VASc score, was higher in fallers compared with nonfallers, on average, by 1 point. This finding underscores the potential benefit of anticoagulation in this population despite the elevated risk of falls. A modeling study estimated that a patient with AF on an anticoagulant would need to fall approximately 295 times per year for the benefits of ischemic stroke prevention with OACs to be outweighed by the potential for serious bleeding [9]. In our study, traumatic bleeding was responsible for half of bleeding episodes, and recurrent fallers exhibited a higher rate of trauma-related bleeding episodes compared with nonrecurrent fallers. These findings highlight the importance of addressing potentially modifiable bleeding risk factors in patients anticoagulated for AF. As emphasized in the 2024 European Society of Cardiology (ESC) guidelines for the management of AF, regular assessments of bleeding risk, including fall risk, should be incorporated into routine care for these patients [13]. It is also important to recognize that frequent falling is not only a risk factor for bleeding but also for fractures and fear of falling, leading to postfall syndrome and subsequent deconditioning [22,23]. These multiple risks highlight the importance of preventive strategies to decrease the risk of falling in all individuals.

### 4.1 Subgroup analyses

Our subgroup analyses showed that the adjusted risk of death in recurrent fallers compared with nonrecurrent fallers was higher among patients treated with VKAs than those treated with DOACs. Although recurrent fallers treated with VKAs did not have a higher risk of major bleeding, they did have a higher risk of any bleeding, any traumatic bleeding, and death after multivariable adjustment. It is, therefore, possible that bleeding could contribute to mortality in the VKA group. This finding could also reflect the older age and greater comorbidity burden of these patients, with residual confounders not fully accounted for in the multivariable model.

In the present cohort, most patients with AF were treated with DOACs (56% vs 44% taking VKAs), demonstrating their widespread adoption in accordance with the 2024 ESC guidelines for the management of AF [13]. Evidence from a meta-analysis of 13 randomized controlled trials and 27 observational studies supports the effectiveness and safety of DOACs compared with VKAs [24]. While limited data exist specifically for high-fall-risk patients, a randomized trial reported that edoxaban resulted in a greater absolute risk reduction in severe bleeding and all-cause mortality than warfarin in patients at high risk of falls [25]. Similarly, 2 studies found that apixaban resulted in lower risk of intracranial hemorrhage compared with warfarin in this population [26,27]. Our study complements these findings, demonstrating no increased risk of bleeding in recurrent fallers treated with DOACs, whereas recurrent fallers on VKAs had a higher risk of bleeding. These findings further support the preferential use of DOACs in high-fall-risk populations.

### 4.2 Strengths and limitations

Our study has several strengths. First, we used prospective “real-world” data from a large, multicenter cohort of patients with AF treated with DOACs and VKAs. This contrasts with prior studies, which included only a limited number of recurrent fallers receiving anticoagulants, mostly VKAs [4,20,21]. Second, contrary to prior studies that evaluated falls based on chart review criteria, we prospectively identified patients with a history of falls by 1 standardized question, and we focused our study on the objective history of recurrent falls. Therefore, we studied a more specific population, which strengthened our ability to evaluate the potential association between recurrent falls and the risk of bleeding.

Our study also has some limitations. The cohort is composed of patients recruited in Switzerland, and only 27.5% of patients were female, which could limit the external validity of the results. Recurrent fallers were screened based on a self-reported questionnaire, which could have led to an underestimation of recurrent faller incidence. Furthermore, neither the exact number of falls nor medication adherence was recorded during the observation period. However, this method mirrors clinical practice, making our findings highly applicable to real-world settings. Finally, our study was conducted prior to the release of the 2024 ESC guidelines for the management of AF. Consequently, we used the CHA_2_DS_2_-VASc score, which has since been replaced by the Congestive heart failure, Hypertension, Age ≥75 years, Diabetes mellitus, Stroke or transient ischemic attack, Vascular disease, Age 65–74 years (CHA_2_DS_2_-VA) score [13].

## 5 Conclusion

Our study found an increase in traumatic bleeding episodes but no increased risk of major or minor bleeding in recurrent fallers with AF anticoagulated with DOACs or VKAs. Our results support the anticoagulation of patients with a high thromboembolic risk but also emphasize the importance of addressing potentially modifiable bleeding risk factors in patients anticoagulated for AF, as recently suggested in the 2024 ESC guidelines for the management of AF.
